# Supplementary material for: The causal relationship between the human gut microbiota and pyogenic arthritis: a Mendelian randomization study
Source: Front Cell Infect Microbiol. 2024 Nov 26;14:1452480. doi: 10.3389/fcimb.2024.1452480 (PMC11629706; doi:10.3389/fcimb.2024.1452480)
Supplement: Supplementary file 1 [file DataSheet1.zip › mendelian test/ebi-a-GCST90027635.csv_forest.pdf]

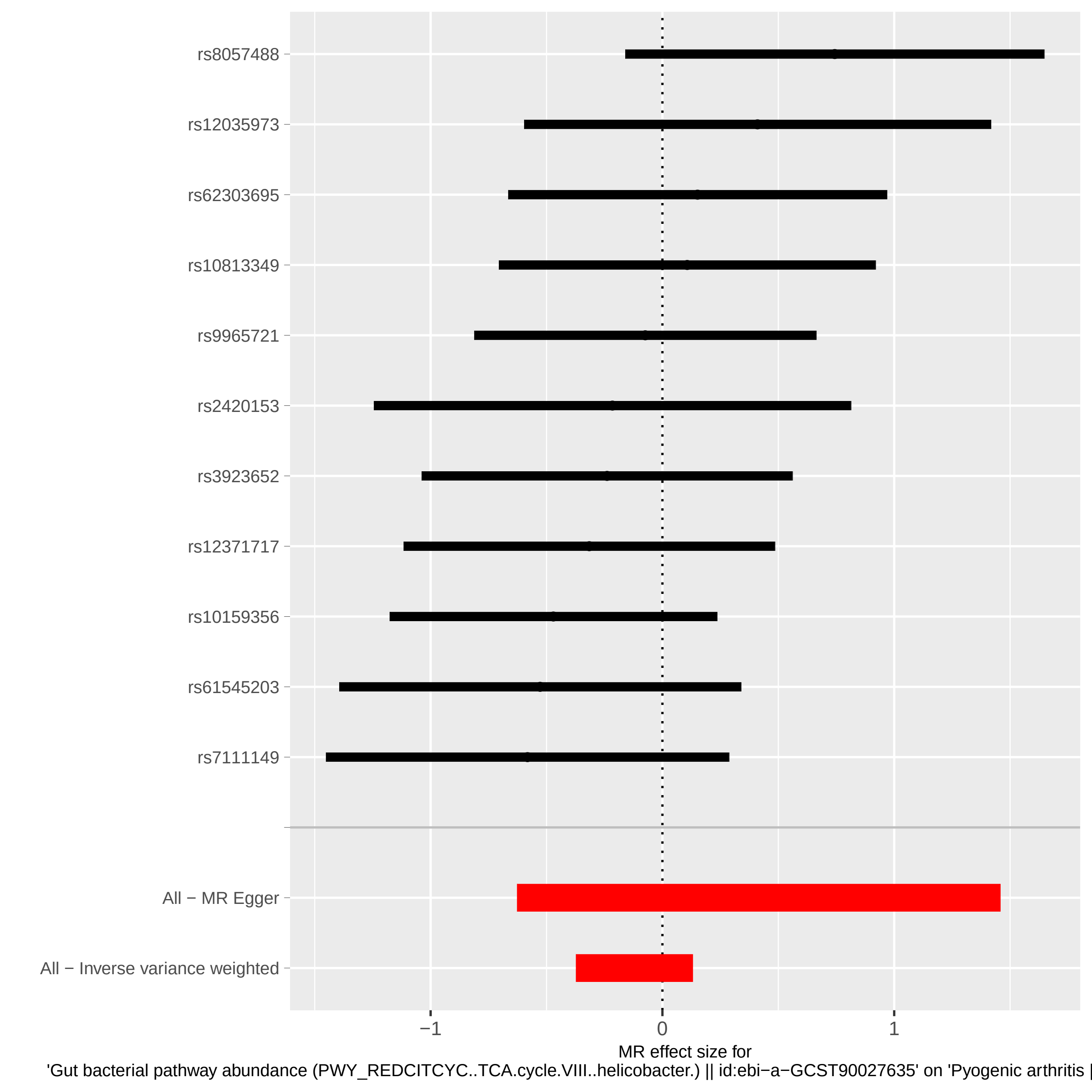
rs8057488
rs12035973
rs62303695
rs10813349
rs9965721
rs2420153
rs3923652
rs12371717
rs10159356
rs61545203
rs7111149
All − MR Egger
All − Inverse variance weighted
−1
0
1
MR effect size for
'Gut bacterial pathway abundance (PWY_REDCITCYC..TCA.cycle.VIII..helicobacter.) || id:ebi−a−GCST90027635' on 'Pyogenic arthritis |
